# Treatment of HNSC and pulmonary metastasis using the anti-helminthic drug niclosamide to modulate Stat3 signaling activity

**DOI:** 10.7150/jca.95682

**Published:** 2024-06-11

**Authors:** Wanjin Jiang, Xiaonan Yang, Xiao Han, Ruijia Gan, Hongting Hua, Dongyu Si, Fuqin Sun, Zhimin Ding, Xinbei Zhu, Qi Yang, Huabing Zhang, Chaobing Gao

**Affiliations:** 1 Department of Otorhinolaryngology Head and Neck Surgery, The First Affiliated Hospital of Anhui Medical University, Hefei, 230022, China.; 2 Department of Otorhinolaryngology Head and Neck Surgery, The First Affiliated Hospital of Wannan Medical College, Wuhu, 241000, China.; 3 Department of Biochemistry & Molecular Biology, Metabolic Disease Research Center, School of Basic Medicine, Anhui Medical University, Hefei, 230032, China.; 4 Department of Gastroenterology, The First Affiliated Hospital of Wannan Medical College, Wuhu, 241000, China.; 5 Department of Otorhinolaryngology Head and Neck Surgery, Xuancheng People's Hospital, Xuancheng, 242000, China.; 6 Department of Otorhinolaryngology Head and Neck Surgery, The Second People's Hospital of Hefei, Hefei, 230011, China.

**Keywords:** Head and neck squamous cell carcinoma, niclosamide, Stat3, β-catenin, apoptosis

## Abstract

**Background:** Head and neck squamous cell carcinoma (HNSC) is a dangerous cancer that represents an important threat to human health. Niclosamide is an anti-helminthic drug that has received FDA approval. In drug repurposing screens, niclosamide was found to inhibit proliferative activity for a range of tumor types. Its functional effects in HNSC, however, have yet to be established.

**Methods:** MTT and colony formation assays were used to explore the impact of niclosamide on the proliferation of HNSC cells, while wound healing and Transwell assays were employed to assess migration and invasivity. Flow cytometry and Western immunoblotting were respectively used to assess cellular apoptosis and protein expression patterns. An HNSC xenograft tumor model system was used to evaluate the *in vivo* antitumor activity of niclosamide, and immunofluorescent staining was employed to assess cleaved Caspase3 and Ki67 expression. The ability of niclosamide to prevent metastatic progression *in vivo* was assessed with a model of pulmonary metastasis.

**Results:** These analyses revealed the ability of niclosamide to suppress HNSC cell migration, proliferation, and invasivity *in vitro* while promoting apoptotic death. From a mechanistic perspective, this drug suppressed Stat3 phosphorylation and β-catenin expression, while increasing cleaved Caspase3 levels in HNSC cells and reducing Bcl-2 levels. Importantly, this drug was able to suppress *in vivo* tumor growth and pulmonary metastasis formation, with immunofluorescent staining confirming that it reduced Ki67 levels and increased cleaved Caspase3 content.

**Conclusion:** In conclusion, these analyses highlight the ability of niclosamide to inhibit HNSC cell migration and proliferative activity while provoking apoptotic death mediated via p-Stat3 and β-catenin pathway inactivation. Niclosamide thus holds promise for repurposing as a candidate drug for the more effective clinical management of HNSC.

## Introduction

Head and neck squamous cell carcinoma (HNSC) is the sixth most prevalent cancer type globally, with an estimated 900,000 diagnoses and 400,000 deaths each year 1. HNSC incidence rates continue to rise and are forecasted to increase by ~30% by 2030 such that this cancer represents a persistent threat to human survival 2. The primary risk factors associated with HNSC incidence include the use of alcohol and tobacco, viral infections, and exposure to certain pollutants in the environment 3. The process through which HNSC tumors develop, however, entails multiple steps and a range of biological factors and pathways including the Stat3, mTOR, NF-κB, Wnt/β-catenin, and Notch signaling pathways 4-6. The 5-year survival rates for individuals diagnosed with HNSC have been improved through advances in multimodal treatment strategies consisting of surgery, chemotherapy, radiotherapy, and/or targeted immunotherapeutic interventions 7,8. Many patients, however, fail to achieve satisfactory therapeutic effects and present with resistance to traditional chemotherapeutic regimens 9. There thus persists an urgent need to identify novel drugs suitable for HNSC patient treatment.

Drug repurposing strategies focus on identifying novel uses for investigational or approved medications that are beyond the scope of their original or established indications 10. As the pharmacokinetic, pharmacodynamic, and toxicity profiles of these drugs are already established, repurposing efforts hold clear advantages over traditional drug development strategies 11. A growing number of research teams have thus began exploring repurposing as a means of identifying new cancer treatments while reducing development times and maintaining lower costs and greater safety 12.

**Figure 1:**
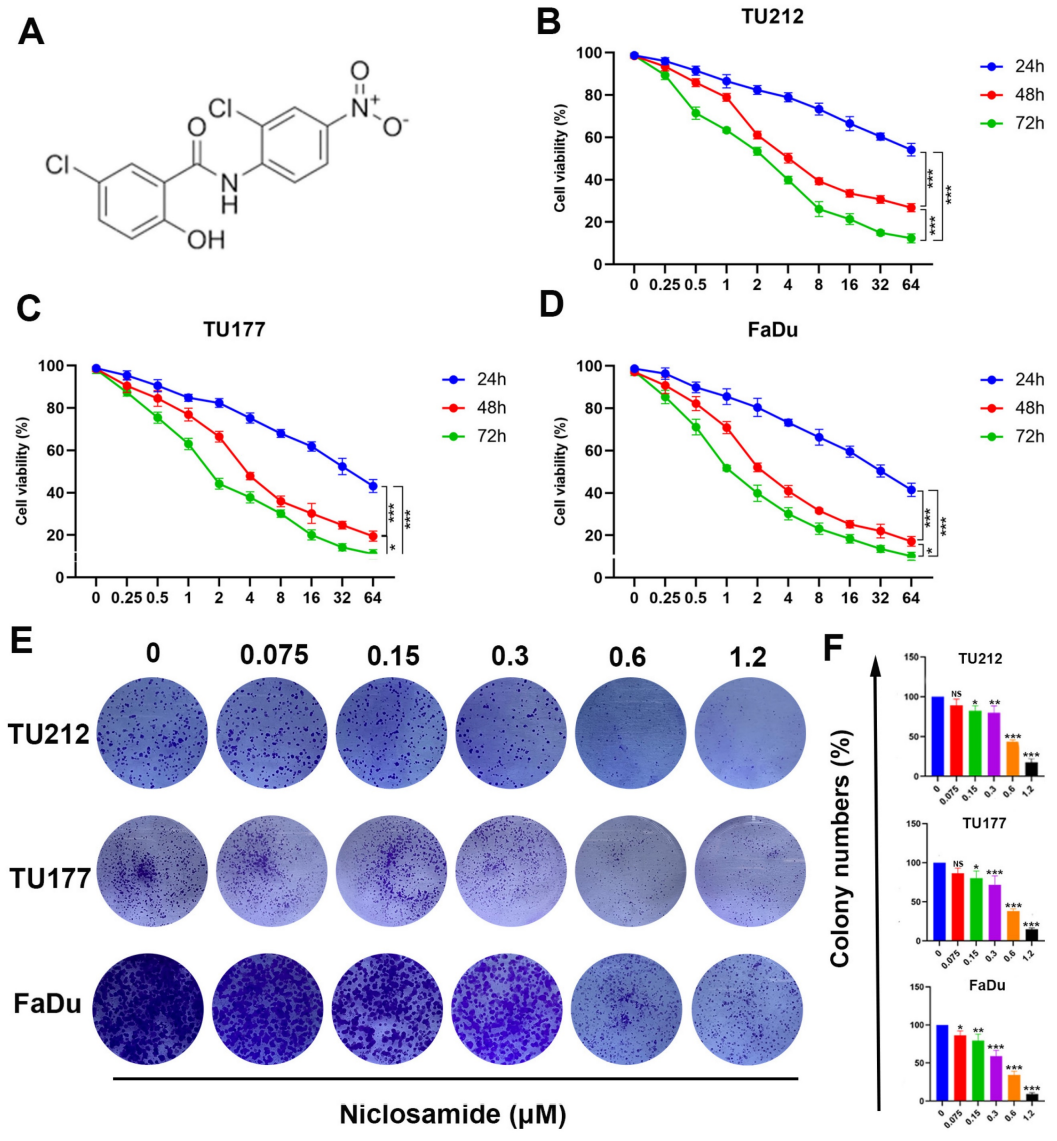
Niclosamide suppresses HNSC tumor cell proliferative activity and viability. (A) Niclosamide chemical structure. (B-D) TU177, TU212, and FaDu cells were treated for 24, 48, or 72 h with various niclosamide concentrations. (n=3). (E) Colony formation activity for niclosamide-treated HNSC cells after 14 days. (n=3). (F) Colony numbers in the indicated groups from (E) (means ± SD). When normalizing experimental results, control treatments were set at 100%. ns: no significance, *P < 0.05, **P < 0.01, ***P < 0.001 vs. control.

The anti-helminthic drug niclosamide (Fig. 1A) has received approval from the US Food and Drug Administration (FDA) and has been used for the treatment of tapeworm infections for almost 50 years 13. More recently, drug repurposing screens and supporting clinical work have revealed that niclosamide also exhibits multipotent biological effects in diseases including cancers, metabolic disorders, and conditions of the respiratory and nervous systems. Indeed, it has been demonstrated to exert a range of antibacterial, anti-inflammatory, antitumor, antifibrotic, and metabolism-regulating activities 14. In mouse models of type 2 diabetes, for example, niclosamide can partially restore normal systemic glucose metabolism 15, while it also inhibits mucus production and airway constriction such that it may be well-suited to the treatment of asthma and pulmonary fibrosis 16. In murine microglia, niclosamide has also been demonstrated to inhibit mTOR and NF-κB signaling activity to abrogate neuro-inflammation 17. Low niclosamide concentrations can suppress the *in vitro* growth of a range of Gram-positive bacteria 18, and there is even evidence that this drug can interfere with the cellular entry and replication of a range of viruses, including the SARS-CoV-2 virus responsible for the COVID-19 pandemic 19,20. Several mechanisms have also been described through which niclosamide can reportedly interfere with the growth and survival of colon, breast, lung, liver, prostate, and ovarian tumors, including its ability to suppress proliferative activity, inhibit cell cycle progression, prevent invasivity and metastasis, and promote apoptosis 21,22. Niclosamide also inhibited the activity of tumor stem cells, and had no significant toxicity to normal cells and non-malignant cancer cells 14. The combination of niclosamide with radiotherapy, chemotherapy, targeted therapy and immunotherapy can increase the drug sensitivity and further improve the anti-tumor effect 22. Few studies to date, however, have examined the effects of niclosamide treatment in HNSC.

Here, niclosamide was found to suppress the proliferation and invasivity of HNSC cells while inducing their apoptotic death via modulating the Stat3/β-catenin signaling. These results highlight niclosamide as a promising candidate drug for HNSC management.

## Materials and methods

### Reagents and antibodies

Niclosamide (99.90% pure), polyethylene glycol 300 (PEG300), and Tween-80 was from MedChemExpress (USA). 3-(4,5-dimethylthiazol-2-yl)-2,5-diphenyltetrazolium bromide (MTT) and dimethyl sulfoxide (DMSO) were from Solarbio (China). For *in vitro* analyses, niclosamide was resuspended in DMSO at 10 mM, followed by storage at -20°C, and dilution with appropriate medium. For *in vivo* analyses, niclosamide was resuspended using 40% PEG300, 5% Tween-80, and 55% saline. An annexin V-FITC/PI apoptosis kit was purchased from Bestbio (China). Antibodies specific for Stat3, p-Stat3, β-catenin, Bcl-2, and β-actin were from Cell Signaling Technology. In addition, antibodies specific for CDK4 (Proteintech), Ki67 (Sigma), cleaved Caspase3 (Abclonal), and β-actin (Santa) were purchased. Beyotime was the source of electrochemiluminescence (ECL) Western blotting detection reagents.

### Cell culture

The human TU212, TU177, and FaDu HNSC cell lines were obtained from the American Type Culture Collection. These cells were grown in DMEM (Gibco) containing fetal bovine serum (FBS, Gibco) and penicillin/streptomycin (Biosharp, China) in a humidified 5% CO_2_ 37°C incubator.

### Cell viability assay

Cells were added to 96-well plates (2x10^3^/well) and allowed to attach, followed by exposure to a range of niclosamide concentrations for 24, 48, or 72 h. Then, MTT solution (25 μL) was added into each well for 1 h, followed by the addition of 100 μL of DMSO for 5 min. Absorbance at 490 nm was then assessed with a microplate reader.

### Colony formation assay

Cells were added to 6-well plates (1x10^3^/well) and exposed to a range of niclosamide concentrations following adherence to the plate. Media was exchanged every 3 days for 14 days, after which methanol was used to fix colonies for 15 min, and 0.1% crystal violet (Beyotime Biotechnology, China) was used for staining at room temperature. Plates were then rinsed twice using PBS, followed by imaging.

### Wound healing assay

Cells were added to 6-well plates (5x10^5^/well) and scratched after adhering to the plate, and then exposed to a range of niclosamide concentrations. Cell migration was assessed after 24 h using an inverted microscope, and ImageJ was used to quantify migratory activity.

### Transwell assay

The upper portion of Transwell inserts was loaded with 200 μL of serum-free medium containing various niclosamide doses and 5x10^4^ HNSC cells, while 600 μL of media with 20% FBS was added into the lower chamber. Following a 24 h incubation period, 4% paraformaldehyde was used to fix invasive cells at room temperature, followed by staining for 30 min using 0.05% crystal violet. Wash the chamber twice with PBS. After cleaning, gently wipe the non-migrated cells in the upper part of the chamber with a cotton swab. Cells were then imaged and counted using an inverted microscope.

### Apoptosis analyses

Cells were added to 6-well plates (5x10^5^/well) and exposed to a range of niclosamide concentrations for 24 h following adherence to the plate. Annexin V binding buffer was then used to resuspend cells, followed by staining using Annexin V-FITC and propodium iodide (PI), after which apoptotic activity was assessed via flow cytometry.

### Western immunoblotting

RIPA buffer (Beyotime) was utilized to extract total cellular protein, followed by SDS-PAGE separation and transfer onto PVDF membranes. Following incubation with appropriate primary and secondary antibodies, ECL reagents were used to detect protein bands with a chemiluminescence apparatus.

### *In vivo* tumor model systems

Mouse model studies received approval from the Animal Ethics Committee of Anhui Medical University, and were performed as per the guidelines established by the Animal Center of Anhui Medical University. For these experiments, male BALB/c nude mice (5 weeks old) from the Model Animal Research Center of Nanjing University were used. To establish xenograft tumors, mice were subcutaneously implanted implanted with 2x10^6^ FaDu cells in a 100 μL volume. When tumors were ~100 mm^3^ in size, mice were randomized into vehicle and niclosamide treatment groups (n=6/group). Niclosamide or vehicle control were administered to these mice once daily (i.p.) for 28 days, measuring body weight and tumor volume every three days. After this 28-day period, mice were euthanized and tumors were harvested for downstream analyses.

For pulmonary metastasis experiments, mice 5x10^6^ FaDu cells in 100 μL were injected via the tail vein, and 6 days later, mice were randomized into two groups (n=4/group) that received i.p. injections of niclosamide or vehicle every other day for 8 weeks, after which mice were euthanized and evaluated.

### Immunofluorescence and histopathology

Tumor tissue samples were fixed using 10% formalin, paraffin-embedded, and cut to generate 5 μm sections. Following antigen repair, 5% goat serum was used to block samples for 30 min at 37°C, followed by overnight incubation with anti-Ki67 or anti-cleaved Caspase3 at 4°C. Sections were then rinsed, probed for 1 h at room temperature using secondary antibodies, and counterstained for 10 min with DAPI. An immunofluorescent microscope was then employed for sample imaging.

Histopathological analyses were performed by fixing, embedding, and sectioning tumor tissues as above. Then, hematoxylin and eosin (H&E) were used to stain these tissue sections, followed by microscopic imaging.

### Statistical analyses

*In vitro* analyses were performed in triplicate. Data are means ± SD and were compared with GraphPad Prism 9.0 using Student's t-tests or one-way ANOVAs. ns: no significance, *P<0.05, **P<0.01, ***P<0.001.

## Results

### Niclosamide inhibits *in vitro* HNSC cell proliferative activity

Niclosamide has previously been demonstrated to suppress the growth of some tumor types 16,21. In an effort to clarify the impacts of niclosamide on HNSC cell proliferation, MTT and colony formation assays were conducted to gauge changes in the viability of the TU212, TU177, and FaDu HNSC cell lines. Niclosamide ultimately suppressed HNSC cell viability in a dose-dependent manner following treatment for 24, 48, or 72 h (Fig. 1B-D), outperforming equivalent concentrations of 5-fluorouracil (Fig. S1). These data were further confirmed through colony formation assays wherein niclosamide suppressed proliferation in a dose-dependent fashion (Fig. 1E-F), highlighting the dose- and time-dependent inhibitory impact of niclosamide administration on the viability of HNSC cells.

### Niclosamide suppresses *in vitro* HNSC cell migratory and invasive activity

**Figure 2:**
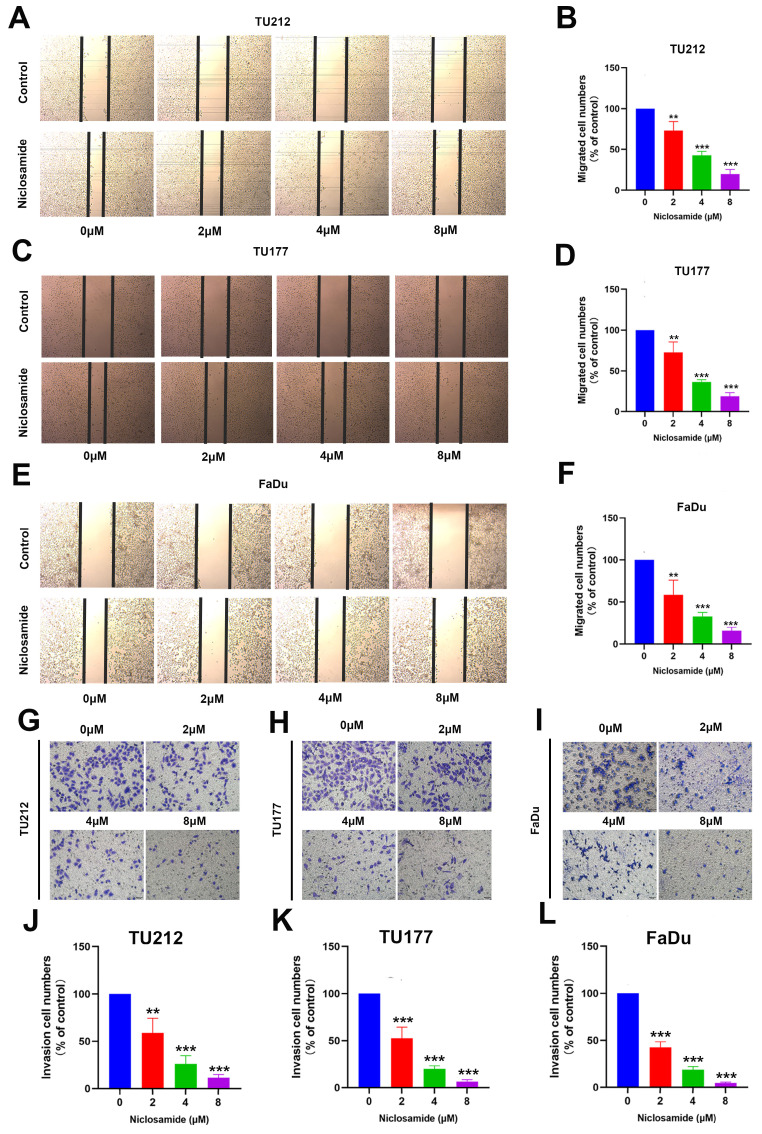
Niclosamide treatment inhibits HNSC cell invasivity and migration. (A-F) Wound healing assays revealing the niclosamide-mediated suppression of migration after 24 hours of niclosamide action. (n=3). (Scale bar: 200μm). (G-L) Transwell assay results revealing niclosamide-mediated suppression of HNSC cell invasivity after 24 hours of niclosamide action. (n=3). (Scale bar: 50μm). **P < 0.01, ***P < 0.001 vs. control.

The growth and metastatic progression of HNSC and other tumor types are closely tied to the migratory and invasive activity of these cells. Accordingly, wound healing and Transwell assays were used to evaluate TU212, TU177, and FaDu cell migration and invasivity in response to niclosamide administration. Wound healing assays indicated that niclosamide was sufficient to significantly suppress the migratory activity of these cells in a dose-dependent fashion after 24 hours of niclosamide action (Fig. 2A-F). Consistently, niclosamide was able to suppress invasivity as compared to control treatment after 24 hours of niclosamide action (Fig. 2G-L). These data offer clear evidence for the ability of niclosamide to suppress *in vitro* HNSC cell migratory and invasive phenotypes.

### Niclosamide promotes HNSC cell apoptotic death

Cells in the early and late stages of apoptosis included 4.6% of control TU212 cells, while this proportion rose to 9.3%, 21.2%, 30%, and 37.1% following treatment with niclosamide at respective doses of 1, 2, 4, and 8 μM for 24 h. In line with these results, the frequency of apoptotic TU177 cells rose from 6.7% in the control group to 16.5%, 23%, 30.1%, and 40.9% in these respective niclosamide treatment groups, with corresponding increases from 6.8% to 16.2%, 27.5%, 37.7%, and 47.7% for FaDu cells. These flow cytometry results offer strong support for the ability of niclosamide to promote the apoptosis of HNSC cells in a dose-dependent fashion.

### Niclosamide suppresses p-Stat3 and β-catenin levels within HNSC cells

**Figure 3:**
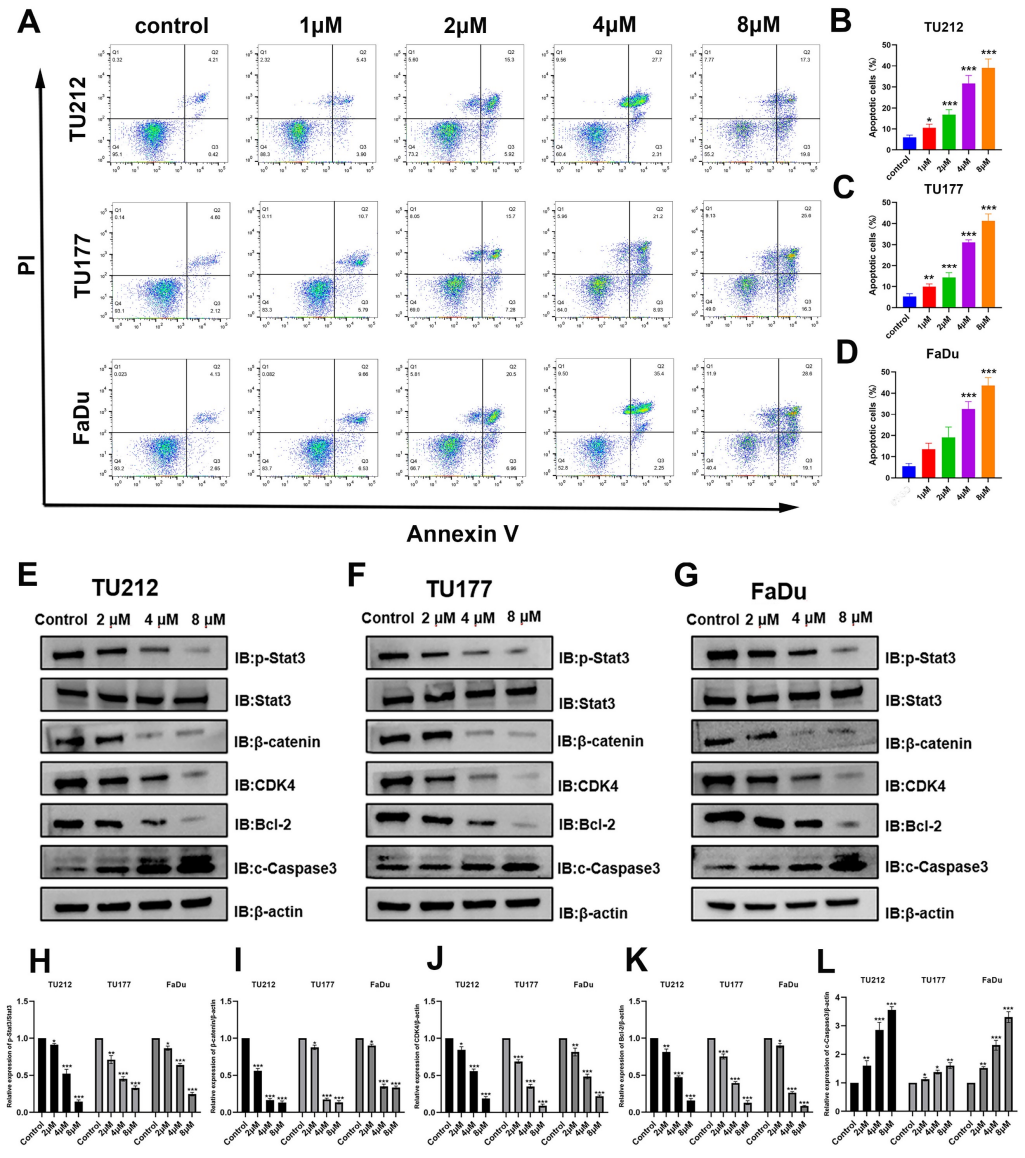
Niclosamide promotes apoptotic HNSC cell death. (A-D) Flow cytometry was used to quantify the apoptotic death of HNSC cells following niclosamide treatment for 24 h. (n=3). (E-L) Levels of proteins including p-Stat3, Stat3, β-catenin, CDK4, Bcl-2, c-Caspase3, and β-actin were assessed following exposure to various niclosamide concentrations for 24 h. (n=3). *P < 0.05, **P < 0.01, ***P < 0.001 vs. control.

These results offer clear evidence of the ability of niclosamide to suppress HNSC cell migratory, invasive, and proliferative activity *in vitro.* To determine whether the expression of proteins in pathways associated with these activities was altered in response to niclosamide administration, Western immunoblotting was next performed (Fig. 3E-L). β-catenin and Stat3 protein signaling are particularly important regulators of tumor onset and progression 23,24. These experiments revealed that niclosamide treatment reduced levels of p-Stat3 and β-catenin within all three tested HNSC cell lines. When assessing apoptosis-associated proteins, pro-apoptotic cleaved Caspase3 levels were significantly higher in all three tested HNSC cell lines after niclosamide treatment. Stat3 signaling activity has been shown to inhibit CKD4 expression while upregulating anti-apoptotic genes such as Bcl-2 25. Consistent changes in CDK4 and Bcl-2 protein levels were detected in niclosamide-treated HNSC cells. These findings thus indicate that niclosamide is capable of inhibiting HNSC cell proliferation and metastatic progression, promoting apoptotic death in part via the inhibition of the Stat3 and β-catenin pathways.

### Niclosamide inhibits *in vivo* HNSC tumor growth

**Figure 4:**
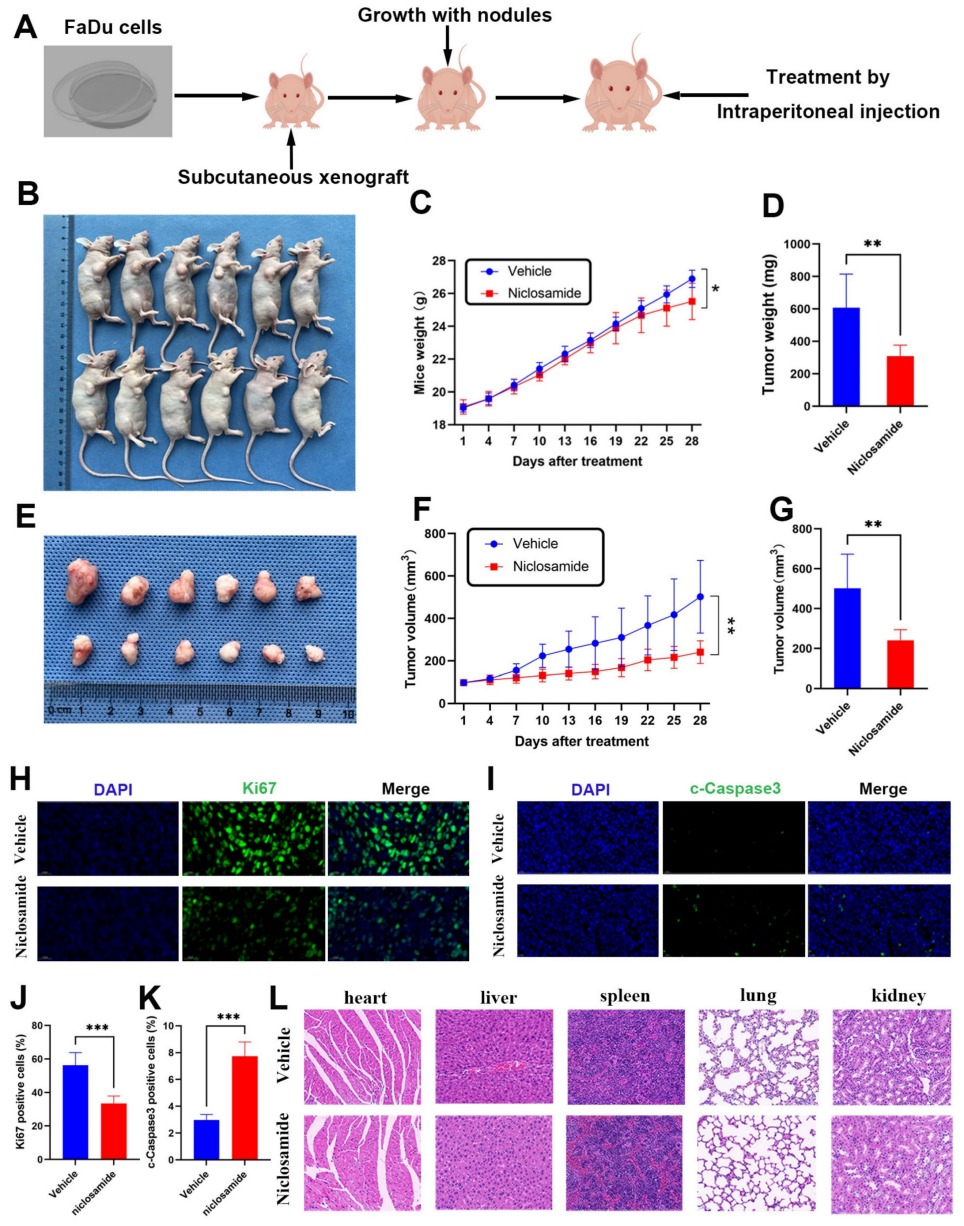
Niclosamide inhibits *in vivo* tumor growth. (A) FaDu xenograft tumor model establishment and treatment approach, with mice being injected with vehicle control or niclosamide for 28 days at a 10 mg/kg dose. (B) Tumors in niclosamide-treated mice on day 28. (n=6). (C) Murine body weight over time. (D) Tumor weights at study end. (E) Tumor images. (F) Tumor volumes at various time points. (G) Tumor volumes at study end. (H-K) Immunofluorescent staining and statistical analyses corresponding to the expression of c- caspase3 and Ki67 in tumor tissues (Scale bar: 20 μm). (L) Hematoxylin and eosin were used to stain histopathological sections (Scale bar: 20 μm). *P < 0.05, **P < 0.01, ***P < 0.001 vs. vehicle.

To extend the above analyses into an *in vivo* setting, BALB/c nude mice were subcutaneously implanted with FaDu cells to establish a xenograft tumor model (Fig. 4A). Relative to the vehicle group, niclosamide treatment was associated with significant reductions in FaDu tumor size (Fig. 4B), with consistent reductions in the weight and volume of these tumors after 28 days (Fig. 4C-E). Specifically, relative to vehicle control, treatment with a 10 mg/kg dose of niclosamide was associated with a 49.1% reduction in tumor weight (Fig. 4D) and a 52% reduction in tumor volume (Fig. 4F-G). Niclosamide also significantly reduced the numbers of Ki67-positive cells in treated tumors relative to vehicle control, with a corresponding increase in the frequency of cells positive for cleaved Caspase3 (Fig. 4H-K). Pathological samples from different organs revealed no apparent differences between the niclosamide and vehicle control groups (Fig. 4L). Overall, these data highlight the ability of niclosamide to inhibit *in vivo* HNSC tumor cell growth via the suppression of Ki67 expression and the upregulation of cleaved Caspase3.

### Niclosamide protects against the pulmonary metastasis of HNSC

**Figure 5:**
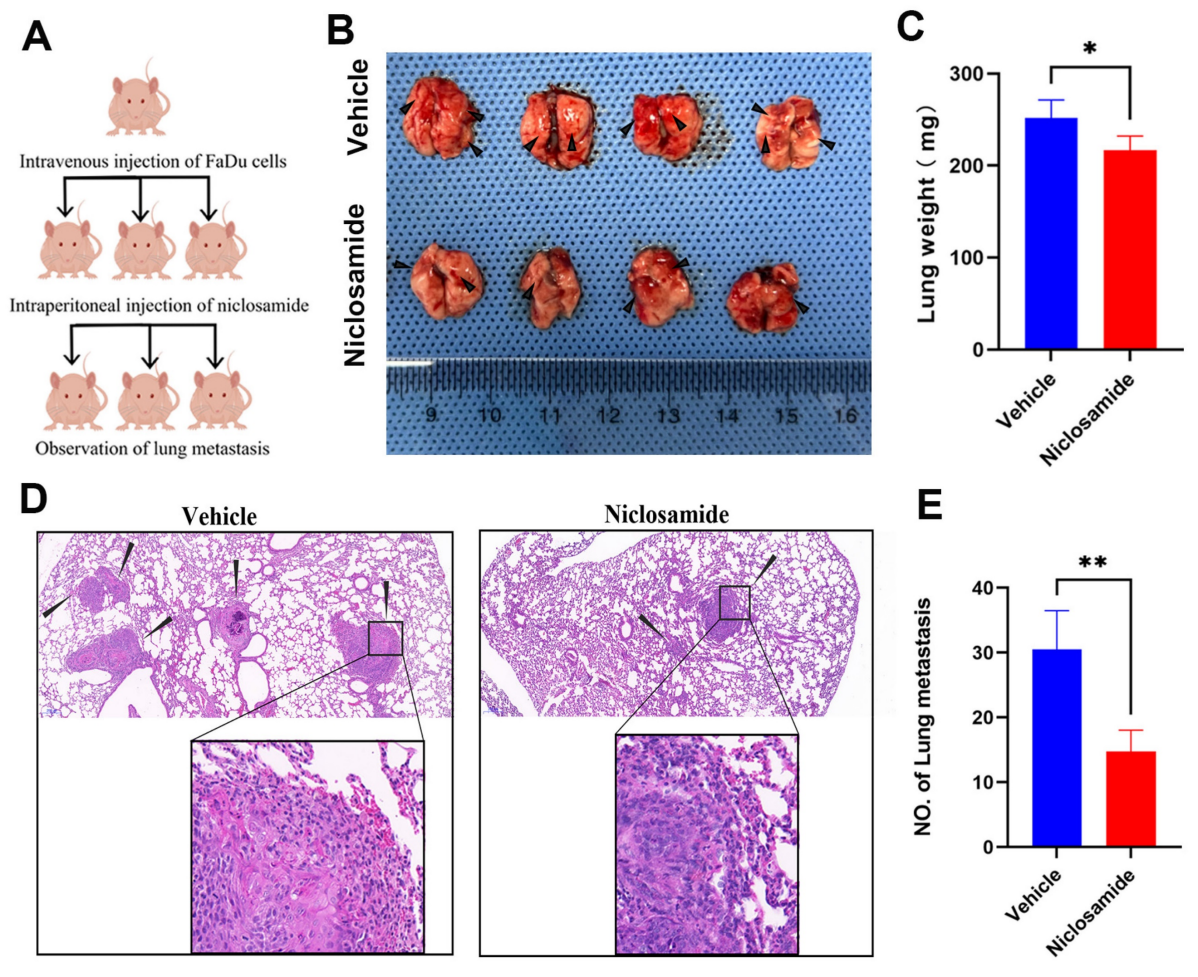
Niclosamide suppresses the pulmonary metastasis of HNSC. (A) Pulmonary metastases were established via injecting 2×10^6^ FaDu cells via the tail vein. (B) Metastatic nodules in the lungs (arrows) of nude mice in the vehicle or niclosamide treatment groups. (n=4). (C) Murine body weight following 8 weeks of niclosamide or vehicle treatment. (D-E) Hematoxylin and eosin staining highlighting pulmonary metastases in the indicated groups (Scale bar: 200 μm and 50 μm). *P < 0.05, **P < 0.01 vs. vehicle.

To more fully clarify the degree to which niclosamide is capable of inhibiting *in vivo* metastatic tumor progression, a pulmonary metastasis model was established by injecting FaDu cells via the tail vein in nude mice (Fig. 5A). As compared to animals in the niclosamide treatment group, a greater increase in lung weight was evident in the vehicle control group (Fig. 5B-C). Consistently, fewer metastatic lesions were evident in the lungs of niclosamide-treated mice (Fig. 5B), as was further confirmed through hematoxylin and eosin staining (Fig. 5D-E). These data highlighted the ability of niclosamide to significantly suppress the pulmonary metastasis of HNSC cells.

## Discussion

HNSC incidence rates continue to rise annually, and despite the assortment of treatment options available to affected patients, rates of late mortality remain high 3. Resistance to conventional chemotherapeutic agents is a common barrier to patient treatment, spurring growing interest in the repurposing of established drugs as a novel approach to cancer management. Niclosamide is an antihelminthic drug that has previously been reported to exhibit antitumor activity mediated through a range of pathways. In a study of ovarian cancer, for example, niclosamide was found to promote apoptotic induction 26, whereas it has also been reported to suppress epithelial-mesenchymal transition induction in breast cancer cells, thereby preventing disease progression 27. Little research on the mechanistic effects of niclosamide on HNSC cells, however, has been published to date.

Here, niclosamide exposure was found to result in the dose- and time-dependent suppression of the proliferation of three HNSC tumor cell lines (TU212, TU177, FaDu), while also preventing these cells from migrating and engaging in invasive activity. Strikingly, niclosamide achieved higher levels of cytotoxicity than equivalent concentrations of the common chemotherapeutic drug 5-fluorouracil. The expression of Stat3 and β-catenin, as well as interactions between the two, has been reported to shape tumor cell invasivity, proliferation, and apoptotic death 23,28. Niclosamide has been shown to suppress Wnt/β-catenin signaling to protect against pulmonary fibrosis 29, while also inhibiting this same pathway to interfere with lung tumor cell growth 30. Wnt/β-catenin pathway regulation and apoptosis have a wide range of functional implications in a variety of diseases 31,32. In this study, while niclosamide inhibited β-catenin protein expression, pro-apoptotic protein cleaved Caspase3 expression was increased, anti-apoptotic protein Bcl-2 expression was decreased, and flow cytometry showed increased apoptosis of tumor cells.

According to these results, niclosamide can inhibit β-catenin expression in HNSC cells and promote the increase of tumor apoptosis. Stat3 is a key transcription factor involved in HNSC oncogenesis and progression, and inhibiting its expression or activity can result in the decreased expression of Bcl-2 and other anti-apoptotic factors 14. Niclosamide has been shown to inhibit liver cancer cell growth while enhancing chemosensitivity via modulating Stat3 signaling 33, and there is also evidence for its ability to suppress prostate tumor cell migration and invasivity through the inhibition of Stat3 pathway activation 34. Consistently, niclosamide treatment reduced Stat3 activation and Bcl-2 expression within HNSC cells. Thus, the inhibition of Wnt/β-catenin and Stat3 signaling pathways by niclosamide resulted in the increase of pro-apoptotic protein and the decrease of anti-apoptotic protein, which led to the increase of apoptosis of tumor cells and inhibited the progression of tumor.

The present results revealed significant decreases in tumor size and weight in niclosamide-treated mice bearing xenograft tumors. Niclosamide has similarly been found to suppress the growth and metastatic progression of thyroid cancer by activating Caspase3 and inhibiting the expression of Bcl-2 35. In line with these findings, cleaved Caspase3 levels were increased following niclosamide administration, while Ki67 levels were decreased. Cleaved caspase-3 is considered to be one of the pro-apoptotic proteins and a reliable marker of apoptosis 36. Ki-67 is a nuclear DNA-binding protein, which functions as a marker of cell proliferation in many cancers, and its high expression often indicates a generally poor clinical prognosis 37. Our experimental results also confirmed that the changes in the levels of these two proteins during the inhibition of tumor development by niclosamide were consistent with previous studies. There is also some evidence for the ability of niclosamide to suppress colon cancer cell growth via inducing the formation of autophagosomes 38,39. As such, this drug may engage a range of context-specific mechanisms to exert its antitumor effects, emphasizing the need for further research focused on these topics. Even so, these data provide clear evidence for niclosamide to induce the apoptotic death of HNSC cells, thereby abrogating tumor growth and progression.

The ability of HNSC cells to readily metastasize is associated with poor prognostic outcomes in affected patients 40. There is prior *in vivo* and *in vitro* evidence for the ability of niclosamide to suppress metastatic progression in various cancers 41. Consistently, niclosamide reduced the burden of lung lesions in a murine HNSC pulmonary metastasis model published in the present study, offering further support for the ability of this drug to protect against *in vivo* metastatic progression.

However, the limitations of the current study should be recognized. The mechanism of action of niclosamide needs further investigation. It is necessary to explore the combination of niclosamide, such as the combination of niclosamide with classical chemotherapy drugs, targeted drugs and immunotherapy drugs to improve the efficacy. Further *in vitro* and clinical trials are needed to confirm the efficacy and safety of niclosamide.

## Conclusion

**Figure 6:**
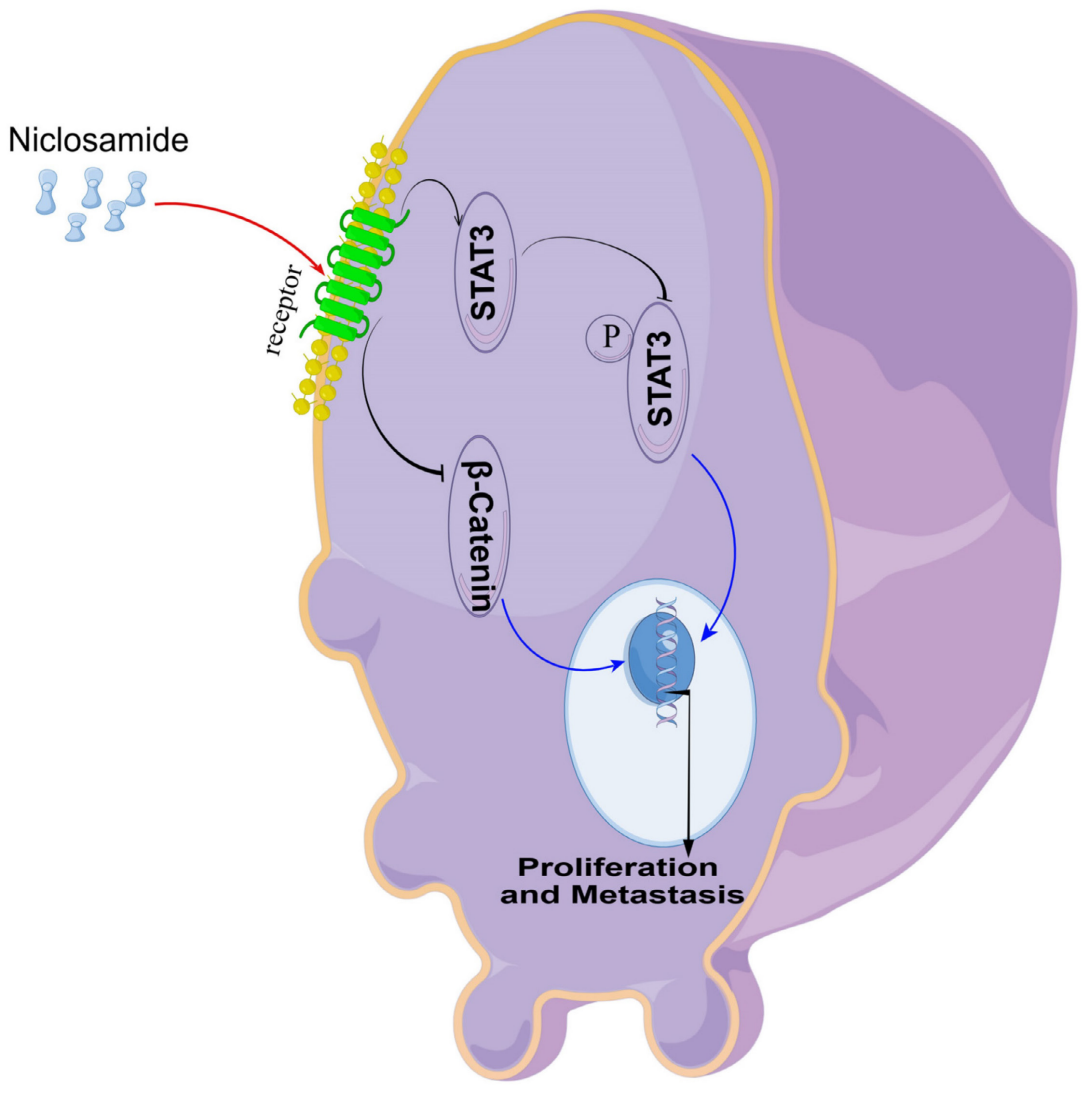
An overview of the proposed mechanism through which niclosamide suppresses the proliferative and metastatic growth of HNSC cells.

*In vitro* experiments confirmed that niclosamide could inhibit the proliferation and invasion of TU212, TU177, and FaDu cell lines, and* in vivo* experiments confirmed that niclosamide could inhibit the proliferation and metastasis of tumor cells in nude mice. In conclusion, the present data offer evidence for the ability of the anti-helminthic drug niclosamide to suppress the *in vitro* and *in vivo* proliferative, migratory, and invasive activity of HNSC cells. Mechanistically, these effects were related to Stat3 and β-catenin pathway inactivation within HNSC cells (Fig. 6). These data underscore the promise of repurposing niclosamide as a candidate strategy for preventing the growth and metastasis of HNSC tumors.

## Supplementary Material

Supplementary figure.

## Abbreviations

HNSC: head and neck squamous cell carcinoma
Stat3: signal transducer and activator of transcription 3
mTOR: mammalian target of rapamycin
NF-κB: nuclear factor kappa-B
PEG: polyethylene glycol
DMSO: dimethyl sulfoxide
CDK: cyclin-dependent kinase
FBS: Fetal bovine serum
5-FU: 5-fluorouracil
PI: propyl iodide
Bcl-2: B-cell lymphoma-2
