# Supplementary figures and images for: Treatment of HNSC and pulmonary metastasis using the anti-helminthic drug niclosamide to modulate Stat3 signaling activity
Source: J Cancer. 2024 Jun 11;15(13):4406–16. doi: 10.7150/jca.95682 (PMC11212102; doi:10.7150/jca.95682)

## Supporting Information

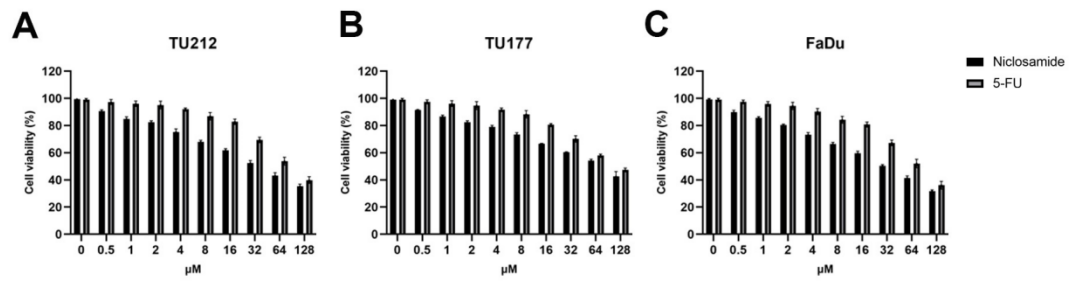

Fig. S1. Effects of niclosamide and 5-FU on HNSC cell lines (TU212, TU177 and FaDu).

Supplement: Supplementary file 1 — Supplementary figure. [file jcav15p4406s1.pdf]
